# Menopausal symptoms, work ability, and quality of life: a cross-sectional study among employed women in southern India

**DOI:** 10.3389/fgwh.2026.1784887

**Published:** 2026-03-25

**Authors:** K. Vidhyashree, Sowmya Koteshwara, B. Sheeba

**Affiliations:** 1 School of Public Health, JSS Medical College, JSS Academy of Higher Education and Research, Mysore, India; 2 Department of OBG, JSS Medical College, JSS Academy of Higher Education and Research, Mysore, India

**Keywords:** employed women, India, menopausal symptoms, menopausal women, menopause rating scale, quality of life, work ability

## Abstract

**Introduction:**

Menopausal symptoms impair midlife women's quality of life and work ability, especially in physically demanding low-resource jobs, yet occupational variations remain underexplored in India. This study examines symptom prevalence, severity, and impacts on work ability and quality of life among southern Indian working women.

**Methods:**

A community-based analytical cross-sectional study was conducted among 280 employed menopausal women aged 45–65 years, representing diverse occupational groups. Data were collected through face-to-face interviews using standardized tools. Correlation analyses and heat-map visualizations examined relationships between symptom severity, quality of life, occupation, and work ability. Bootstrap-adjusted logistic regression with false discovery rate correction identified predictors of poor work ability (WAI ≤27).

**Results:**

Overall, 73.2% of participants reported moderate-to-severe menopausal symptoms, with the highest burden observed among unskilled workers. Symptoms significantly affected physical, psychological, and vasomotor quality-of-life domains, particularly in physically demanding occupations. Women reporting reduced capacity to perform usual activities had nearly twice the odds of poor work ability. Sleep problems and fatigue were also independently associated with poor work ability. Symptom severity showed a strong positive correlation with poorer quality of life and a negative association with work ability; most pronounced among unskilled women.

**Conclusion:**

Menopausal symptoms are highly prevalent and adversely affect work ability and quality of life among employed women, especially those in low-autonomy or physically demanding jobs. These findings underscore the need for occupation-sensitive workplace policies, targeted health education, and supportive interventions to promote well-being, productivity, and workforce retention.

## 1 Introduction

Menopause is a major transitional stage of a woman's life, marked by permanent cessation of menstruation for at least 12 consecutive months due to the loss of ovarian function, and most commonly occurs between the ages of 45 and 55 years worldwide (1, 2). Some women experience menopause as early as 40 years of age – termed as premature or early menopause – or as late as 60 years, known as late menopause. This variation suggests that, in addition to genetic factors, influences from very early life, including lifestyle, environmental, and health-related factors, may affect the age at menopause (3–5).

This natural biological process is accompanied by a decline in hormone production, particularly oestrogen and progesterone, leading to both physiological and psychological changes. The symptoms associated with it can vary widely among women, both in terms of severity and duration (6). Common symptoms include vasomotor disturbances such as hot flashes and night sweats, as well as physical symptoms including fatigue, joint pain, and sleep disturbances (7). These symptoms can impact overall wellbeing of woman, influencing her physical health, emotional well-being, and social relationships. Although menopause is not a medical condition, but a part of the ageing process, it can have profound implications for women's health and quality of life, and may also significantly affect their professional lives by contributing to decreased productivity and increased absenteeism at work (8).

Menopause is a universal experience that affects women globally; an estimated 467 million women are currently postmenopausal, highlighting a substantial and growing demographic group with distinct health needs (9). Globally, joint and muscle pain was found to be the most common menopausal symptom (65.4%), followed by hot flashes which was reported more frequently in low-income (65.9%) than in high-income countries (49.7%). Depression and urogenital issues are especially prevalent in South America, indicating how symptom experiences vary across regions. Regionally, Africa shows the highest overall symptom prevalence, with hot flashes affecting up to 72.6% of women in Egypt, while Oceania had the lowest prevalence (39.9%). In Asia, prevalence varied among ethnic groups, Vietnamese women reported the highest prevalence, while Indonesian women had the lowest (10).

Women in low-income countries face a higher incidence of severe symptoms compared to those in high-income nations (11). The burden of menopausal symptoms is compounded in low and middle-income countries (LMICs), where healthcare resources may be limited, impacting women's access to necessary treatments and support (12). Women with premature ovarian insufficiency (POI) are found to have a twofold higher risk of death from heart disease and a fourfold higher risk of mortality from any cancer compared with women who experience menopause at a later age (13).

Evidence among Norwegian postmenopausal women showed that each additional 3 years of later age at natural menopause was linked to a 1.6% lower all-cause mortality risk. This relationship is particularly pronounced in younger women, suggesting that later onset of menopause may confer protective health benefits (14). Conversely, early onset of menopause (before the age of 45 years) correlates the risk of developing coronary heart disease and overall cardiovascular mortality (15). Menopause is also linked with increased risks of certain cancers, notably breast and endometrial cancers (16).

In India, economic transformation and social change have resulted in growing number of women entering and remaining in the workforce during their menopausal years. Employed women in LMICs face unique challenges, with occupational exposures, workplace stressors, and limited access to supportive policies potentially amplifying the burden of menopausal symptoms and their consequences (17). A secondary data analysis of the Longitudinal Aging Study of India (LASI) reported that about 60% of older adults in their 50s remain employed, with women accounting for about 37% of this working population. Most of these women are engaged in farming or agricultural labourers, fishing, forestry related work (18).

Research indicates that a substantial number of women report experiencing moderate to severe menopausal symptoms that can hinder their ability to perform effectively at work (19). With respect to its impact on job performance, menopausal symptoms can also lead to increased absenteeism (20). Studies also suggest that up to 10% of women aged 45–60 years may take time off work due to menopause-related issues (21).

The stigma surrounding menopause often leads women to feel embarrassed to discuss their symptoms with employers. This lack of communication can create an environment where women feel unsupported, exacerbating feelings of isolation and distress during this menopausal period (22). Cultural norms may dictate how menopause is perceived and discussed within families and workplaces, potentially leading to a lack of awareness of the challenges faced by women during this period. Many women may struggle with societal expectations while simultaneously dealing with physical and emotional changes that are often misunderstood or overlooked (23).

Therefore, this study aims to bridge these gaps by assessing the prevalence and severity of menopausal symptoms among employed women in Southern India, and by examining their association with work ability and quality of life across a range of occupational groups. The results have the potential to inform workplace health policies and supportive strategies to enhance both employee well-being and productivity in this rapidly expanding and aging workforce.

## 2 Materials and methods

### 2.1 Study design and study setting

A community-based analytical cross-sectional study was carried out between June 2024 and June 2025 in the urban and rural field practice areas of a private medical college in Mysore. The selected urban filed practice areas were Bannimantap and Medhar block, while Kadakola and Hadinaru represented the rural settings in the study. Prior written approval was obtained from the District Health Officer (DHO) before initiation of the study. This study was conducted and reported in compliance with the STROBE (Strengthening the Reporting of Observational Studies in Epidemiology) guidelines for observational research.

### 2.2 Sample size

A previous study conducted among middle-aged Indian women reported that the prevalence of menopausal symptoms was 87.7% (21). The sample size for the present study was estimated using the formula *n* = Z^2^ *p* (1-p)/d^2^, setting the confidence interval at 95% and a 4% precision yielding a calculated sample of 254 participants. After adjusting for an anticipated 10% non-response rate, the final sample size was set at 280 participants. A total of 280 women were recruited ensuring equal representation of urban and rural areas. This sample size was considered appropriate given the specific aim of focusing on natural menopausal and working women, particularly in light of the recent upsurge in hysterectomy cases.

### 2.3 Study participants

The study population comprised women aged between 45 and 65 years of age who had attained natural menopause and had been residing in the selected field practice area for more than six months. Eligible participants were those with amenorrhoea for at least 12 months and not more than 10 years post-menopause, who were currently employed either in the organized sector (professional, clerical, skilled occupations) or the unorganized sector (unskilled labour, farming, etc.), and who provided voluntary informed consent to participate. Women were excluded if they had:

- Current use of antidepressants/ antipsychotic drugs
- History of hysterectomy/ oophorectomy
- Diagnosed with any major medical condition or currently using medications impacting quality of life or mimicking menopausal symptoms (e.g., active cancer, uncontrolled thyroid disease, uncontrolled diabetes, hormone replacement therapy).
- Severe mental health diagnosis affecting comprehension and study participation.
- Known cases of autoimmune diseases such as Cushing's syndrome or genital pathology.
- Amenorrhoea in the past 12 months due to other physiological conditions (pregnancy, lactating amenorrhoea etc.).

### 2.4 Sampling technique and data collection

**Figure 1:**
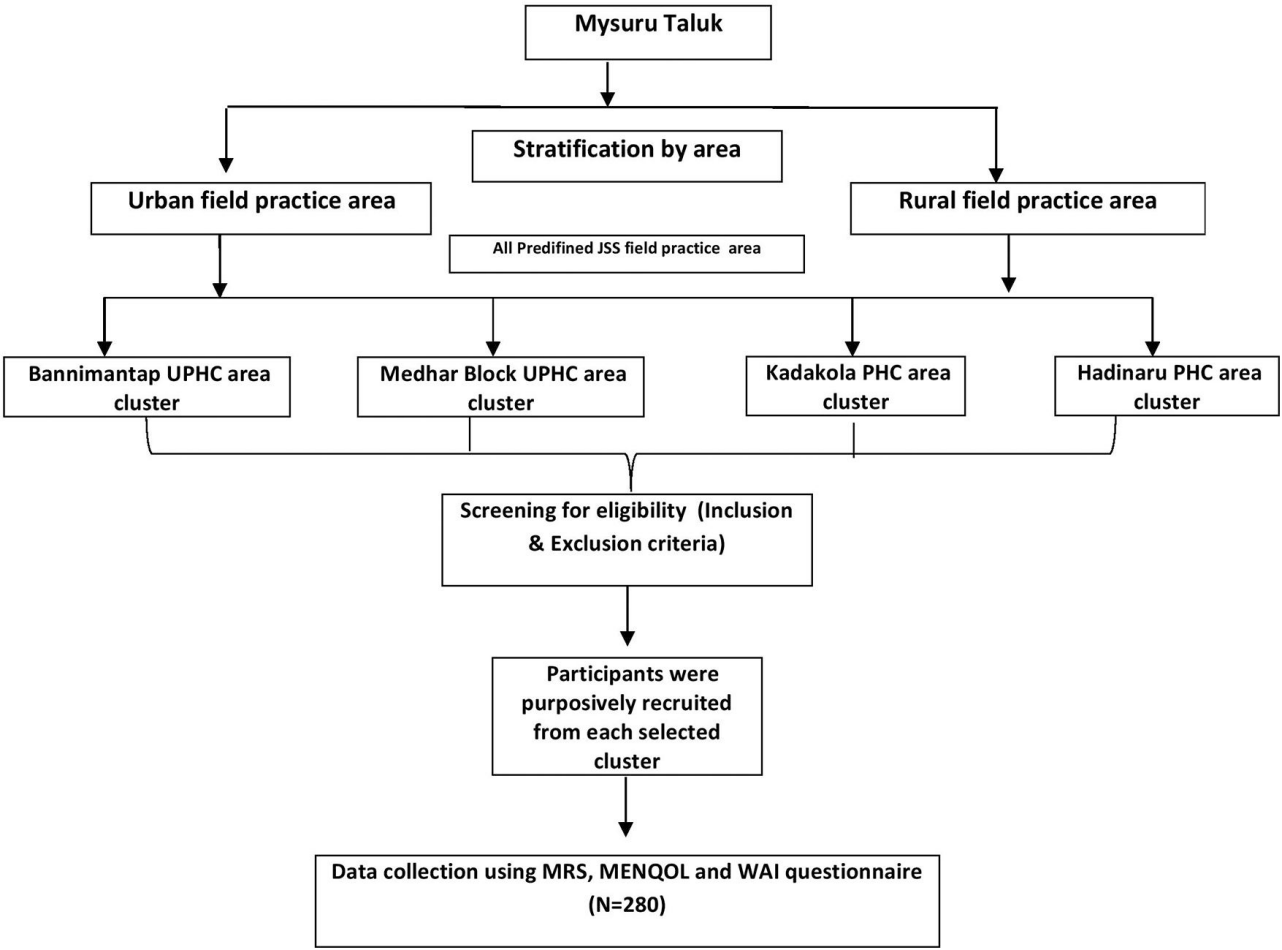
Schematic representation of the study participant recruitment and sampling process.

The study employed a multistage sampling approach. Initially, Mysuru taluk was stratified into urban and rural regions to ensure representation from both settings. Predefined urban and rural field practice areas were considered as clusters and were selected from each stratum. From the urban stratum, two clusters (the Bannimantap Urban Health Centre area and the Medhar Block Urban Primary Health centre area) and from the rural stratum, two clusters (the Kadakola Primary Health Centre area and the Hadinaru Primary Health Centre area) were chosen. From each of these four clusters, 70 participants were planned to be recruited. Within each cluster, participants were recruited using purposive sampling (Figure 1). Purposive final-stage recruitment was chosen for its feasibility in this resource-constrained field scenario targeting employed postmenopausal women—a difficult-to-reach demographic with limited sampling frames. Due to the lack of household registers for informal labourers, probability sampling was not viable.

Eligible women were approached with house-to-house visits, and selection was based on predefined inclusion and exclusion criteria. After eligible participants were briefed on the study purpose and objectives using the participation information sheet (PIS), written informed consent was obtained. Face- to-face interviews were conducted after ensuring thorough confidentiality and privacy. Data collection was carried out using a standardised, pretested interviewer-administered questionnaire. A pilot study involving 20% of the planned sample was conducted before the main study, and the questionnaire was subsequently refined based on the findings. The Menopause-Specific Quality of Life Questionnaire (MENQOL) was used to measure quality of life, the Women's Ability Index (WAI) was used to measure autonomy and decision-making ability, and the Menopause Rating Scale (MRS) was used to measure the severity of menopausal symptoms. To guarantee consistency and data quality, a qualified investigator collected the data under supervision. The questionnaire was interviewer-administered by trained bilingual trained researcher in English and Kannada; however, formal back-translation was not undertaken.

### 2.5 Study variables

#### 2.5.1 Occupation classification

Occupational status was operationalised using the skill level and typical job roles consistent with widely used Indian socioeconomic and labour-classification schemes. Women's current jobs were grouped into four categories:

##### 2.5.1.1 Unskilled workers

Positions entailing basic manual labour that necessitate neither formal education nor specialised training (e.g., daily wage labourers, domestic workers, assistants), consistent with definitions characterising unskilled work as demanding “minimal or no independent judgement or prior experience.”

##### 2.5.1.2 Semi-skilled workers

Roles requiring some training or on-the-job experience to perform routine operations under supervision (e.g., factory or workshop helpers, machine operators, drivers, attendants).

##### 2.5.1.3 Skilled workers

Professions requiring specialised craft skills and significant autonomous judgement, include tailors, electricians, mechanics, healthcare professionals, or other technically skilled personnel.

##### 2.5.1.4 Clerical/farmer/shop owner

This study combined the clerical, shop owner, and farmer categories, consistent with the occupational component of the Kuppuswamy and BG Prasad socioeconomic scales.

##### 2.5.1.5 Professional

Included highly skilled occupations such as teachers, nurses, administrators and other graduates in professional services (24–26).

#### 2.5.2 Menopause symptoms

The Menopause Rating Scale (MRS) was used to assess the menopausal symptoms. It is a standardised instrument that assesses the severity of menopausal symptoms and their impact on a woman's everyday life. It includes 11 symptoms across three domains, namely physical, psychological, and urogenital. Each symptom is rated from 0 (none) to 4 (very severe), with higher total scores showing greater symptom impact. The MRS has been proven reliable and valid, with strong internal consistency (Cronbach's alpha 0.70–0.90) and stable results over time (test-retest reliability ICC 0.70–0.90) and has been validated to be used in India (27).

#### 2.5.3 Menopause-specific quality of life (MENQOL)

The MENQOL questionnaire was used to understand how menopause affects different areas of a woman's overall life, including physical health, emotional well-being, sexual life, and common symptoms like hot flashes. It's a standardized tool that has been widely used. Most women who have used it found the questions clear and relevant, reflected in a high face validity score of 4.7 out of 5. It's also proven to be reliable, with consistent results over time. This questionnaire also does a good job of picking up real changes in women's experiences. Its ability to reflect improvements or worsening of symptoms over time makes it a useful tool for both research and clinical settings. Because of its clarity, accuracy, and sensitivity to change, the MENQOL was chosen as the most suitable tool for this study (28).

#### 2.5.4 Workability

The Work Ability Index (WAI) is a standardised instrument designed to evaluate individuals' capacity to meet employment demands in relation to their physical and mental health. The WAI demonstrates strong internal consistency and reliability, meaning it yields consistent results and has good construct validity, effectively measuring the work ability of an individual. This makes the WAI a credible tool for evaluating workforce health. WAI is valid and reliable in occupational settings, particularly in healthcare. The total score ranges from 7 to 49 (29).

### 2.6 Ethical consideration

The study received approval from the Institutional Ethics Committee (IEC) of the Medical College (JSS/MC/PG/Mph-1/2024-25). The study was carried out following the principles of the Declaration of Helsinki and the 2017 Indian Council of Medical Research (ICMR) guidelines. Participants received a written information sheet, which was read aloud to those with limited literacy before obtaining written informed consent; thumb impressions were recorded from participants who could not sign. Participant confidentiality and privacy were ensured throughout the study, with only de-identified data used for analysis and reporting of results.

### 2.7 Statistical analysis

Data collected using Google Forms were downloaded in the Microsoft Excel format, where preliminary cleaning and coding were performed to ensure accuracy and consistency. The cleaned dataset was then imported into IBM SPSS (Statistical Package for the Social Sciences) statistics version 30 (IBM Corp., Armonk, NY, USA, university licensed) for analysis. Descriptive statistics summarised categorical variables as frequencies and percentages, whereas continuous variables were presented as means and standard deviations. To assess the association between menopausal symptoms, socio-demographic factors, and work-related variables, the chi-square test (*χ*^2^) was applied among categorical variables. Correlation analysis was done to examine association between menopausal symptoms and work ability across occupations, as well as between MRS scores and quality of life by occupation. A correlation matrix was also generated to visualise the relationship between MENQOL domains and with work ability within each occupational category. Furthermore, logistic regression was used to identify factors associated with low WAI scores (≤27) based on the standard cut of score. Given potential violations of normality and model instability, a bootstrap approach with 400 resamples (as the sample size is small) was applied to obtain robust estimates of odds ratios (ORs) and corresponding 95% confidence intervals (CIs). Both sociodemographic variables and menopausal symptoms were included as independent variables (*X*_i_) in the model. To account for multiple comparisons, *p*-values were adjusted using the false discovery rate (FDR) method. Variables with FDR-adjusted *p*-values <0.05 were considered statistically significant. Also, multicollinearity was assessed using variance inflation factors (VIFs). Results are presented as ORs with 95% CIs.

## 3 Results

### 3.1 Socio-demographic characteristics

The study included 280 working women between the ages of 45 and 65 who had experienced a natural menopause and there were no missing data. Most women (61.8%) experienced menopause between the ages of 41 and 46, and 35.7% between 47 and 51 years. Participants were drawn from both urban and rural areas, with 50% emanating from each. The majority of participants identified as Hindu (72.5%), followed by Muslim (13.2%) and Christian (11.1%). Most participants were married (45.4%), with the remainder separated/divorced/widowed (40.7%) or unmarried (13.9%). Nuclear families were most common (46.6%), followed by joint (37.5%) and three-generation families (6.4%). Regarding educational status, 27.9% of the participants were illiterate, 20.7% completed primary school, and 20.7% had middle school education, with the remainder having higher secondary and above. Occupationally, 33.9% were unskilled workers, 15.7% were semi-skilled/skilled, and the remaining participants were in clerical, farmer, shop owner, or professional roles. Socioeconomically, 41.4% belonged to the middle class, 16.8% to the lower middles class, and 11.4% to the upper class (Table 1).

**Table 1:** Sociodemographic characteristics of the study participants (*N* = 280).

Socio-demographic characteristics	Frequency (*n*)	Percentage (%)
Age group		
45–50 years	129	46.1
51–54 years	130	46.4
55 and above	21	7.5
Residence		
Rural	140	50
Urban	140	50
Religion		
Hinduism	222	79.3
Islam	53	18.9
Christianity	5	1.8
Marital status		
Married	149	53.2
Unmarried	5	1.8
Others(separated/divorced/widowed)	126	45
Family type		
Nuclear	148	52.9
Joint	109	38.9
Three generation family	23	8.2
Highest attained qualification		
Illiterate	80	28.6
Primary school	77	27.5
Middle school	42	15.0
High school above	81	28.9
Participant's occupation		
Unskilled	129	46.1
Semi-skilled/Skilled	59	21.1
Clerical, farmer, shop owner, profession	92	32.9
Socioeconomic status		
Upper class	5	1.8
Upper middle class	62	22.1
Middle class	148	52.9
Lower middle class	64	22.0
Lower class	1	0.8

Values are expressed as frequency (*n*) and percentage (%).

Socioeconomic status was assessed using the modified BG Prasad classification updated version.

### 3.2 Prevalence and severity of menopausal symptoms

In this current study, majority (73.2%) of the study participants reported that they experienced moderate to severe symptoms, very few (13.6%) had mild symptoms, and 13.2% reported none. Common somatic symptoms included joint and muscle discomfort (42.9% mild, 36.8% moderate), sleep disturbances (36.4% none, 25.0% mild, 24.3% moderate), and hot flushes/sweating (32.5% mild, 28.2% moderate). Psychological symptoms such as tiredness (42.9% moderate, 30% mild), irritability (40.7% mild), and anxiety (35.7% mild) were present. Urogenital symptoms included vaginal dryness (28.9% mild), bladder problems (38.9% mild, 24.3% moderate), and sexual problems (69.6% none, 17.5% mild).

Furthermore, when analysed by occupation, notable differences in the prevalence and severity of symptoms were reported. Among the 129 individuals who work as unskilled labourers, 46.3% reported moderate to severe symptoms, 11.7% reported mild symptoms, and 10.6% reported no symptoms. Amongst those in semi-skilled or skilled occupations (*n* = 59), 2.1% reported no symptoms, 3.7% reported mild symptoms, and 25.5% reported moderate to severe symptoms. Among those employed as clerical workers, farmers, shop owners and professionals (*n* = 92), 37.2% had moderate to severe symptoms, 4.8% reported mild symptoms, and 6.9% had no symptoms (Table 2).

**Table 2:** Distribution of study participants based on the menopausal symptoms across occupation (*N* = 280).

Menopausal symptoms	Frequency (I)	Percentage (%)
Unskilled (*n* = 129)		
None	20	10.6
Mild	22	11.7
Moderate to severe	27	46.3
Semiskilled and skilled (*n* = 59)		
None	4	2.1
Mild	7	3.7
Moderate to severe	48	25.5
Clerical/Farmer/Shop owner/Profession (*n* = 92)		
None	13	6.9
Mild	9	4.8
Moderate to severe	70	37.2

Menopausal symptoms were assessed using the Menopause Rating Scale (MRS) and categorized as none, mild, and moderate to severe.

Percentages are calculated within each occupational category.

### 3.3 Quality of life

#### 3.3.1 Physical domain

The most common physical symptoms were joint and muscle discomfort (with 36.8% experiencing moderate and 42.9% mild levels), followed by sleeping problems and hot flushes/sweating. Heart discomfort was mostly mild (43.2%). Physical symptoms led to a mild decline in quality of life (QoL) for 77.1% of women, a moderate decline in 13.9%, and no effect in 8.9%. Decline was significantly associated with age group (51–54 years), residing in the urban area, and having lower educational status (*p* < 0.005).

Among unskilled women, 67.4% reported a decline in physical quality of life related to moderate to severe menopausal symptoms; this association is statistically significant (*p* < 0.001). In contrast, semi-skilled/skilled and professional groups showed lower prevalence of physical QoL decline (47.5% and 55.4%, respectively), and these were not statistically significant (Table 3).

**Table 3:** Association between menopausal symptom with MENQOL across occupation (*N* = 280).

Menopausal symptoms	MENQOL domains		Chi square	*p* value
Physical domain				
	No effect (*n*-25)	Decline (*n*-255)		
Unskilled (*n*-129)				
None	12 (9.3)	8 (6.2)	45.16	**0**.000*
Mild	2 (1.6)	20 (15.5)		
Moderate to severe	0 (0)	87 (67.4)		
Semi-skilled & skilled (*n*-59)				
None	1 (1.7)	16 (27.1)	1.452	0.58*
Mild	2 (3.4)	10 (16.9)		
Moderate to severe	2 (3.4)	28 (47.5)		
Clerical/Farmer/Shop owner/Profession(=92)				
None	3 (3.3)	17 (18.5)	2.877	0.24*
Mild	1 (1.7)	18 (19.6)		
Moderate to severe	2 (2.2)	51 (55.4)		
Psychosocial domain				
	No effect (*n*-70)	Decline (*n*-210)		
Unskilled (*n* = 129)				
None	16 (12.4)	4 (3.1)	35.64	**0**.**001**
Mild	5 (3.9)	17 (13.2)		
Moderate to severe	13 (10.1)	74 (57.4)		
Semiskilled and skilled (*n* = 59)				
None	7 (11.9)	10 (16.9)	2.924	0.219*
Mild	2 (3.4)	10 (16.9)		
Moderate to severe	6 (10.2)	24 (40.7)		
Clerical/Farmer/Shop owner/Profession(=92)				
None	4 (4.3)	16 (17.40)	0.302	0.889*
Mild	5 (5.4)	14 (15.2)		
Moderate to severe	12 (13.0)	41 (44.6)		
Vasomotor domain				
	No effect (*n*-79)	Decline (*n*-201)		
Unskilled(*n* = 129)				
None	18 (14.0)	2 (1.6)	38.62	**0**.**001**
Mild	10 (7.8)	12 (9.3)		
Moderate to severe	16 (12.4)	71 (55.0)		
Semi-skilled and skilled (*n* = 59)				
None	5 (8.5)	12 (20.3)	0.638	0.794*
Mild	2 (3.4)	10 (16.9)		
Moderate to severe	8 (13.6)	22 (37.3)		
Clerical/Farmer/Shop owner/Profession (n = 92)				
None	3 (3.3)	17 (18.5)	1.374	0.540*
Mild	3 (3.3)	16 (17.4)		
Moderate to severe	14 (15.2)	39 (42.4)		
Sexual domain				
	No effect (*n*-199)	Decline (*n*-81)		
Unskilled (*n* = 129)				
None	14 (10.9)	6 (4.7)	1.426	0.503
Mild	14 (10.9)	8 (36.4)		
Moderate to severe	66 (51.2)	21 (16.3)		
Semiskilled and skilled (*n* = 59)				
None	9 (15.3)	8 (13.6)	4.983	0.960
Mild	11 (18.6)	1 (1.7)		
Moderate to severe	21 (35.6)	9 (15.3)		
Clerical/Farmer/Shop owner/Profession (=92)				
None	14 (15.2)	6 (6.5)	1.103	0.576
Mild	15 (16.3)	4 (4.3)		
Moderate to severe	35 (38.0)	18 (19.6)		

Association between severity of menopausal symptoms and quality of life across occupational categories was assessed using the Chi-square test.

MENQOL domains include physical, psychosocial, vasomotor, and sexual domains. Percentages are calculated within each occupational group.

Bold values indicate statistically significant results (*p* < 0.05).

* Denotes fishers’ exact values.

#### 3.3.2 Psychosocial domain

A mild decline in psychosocial quality of life was reported by 59.3%, and a moderate decline by 15.7% of women. Significant associations were observed with religion (higher decline in Hindus) and marital status (greater decline among married, separated, or widowed women). A significant decline in psychosocial quality of life was observed in unskilled women with moderate to severe symptoms (57.4%, *p* < 0.001). Although semi-skilled/skilled (40.7%) and professional/clerical groups (44.6%) also reported declines, these were not statistically significant (Table 3).

#### 3.3.3 Vasomotor and sexual domains

Moderate to severe vasomotor symptoms impacted quality of life for 41.4% of women. In the sexual domain, most (71.1%) reported no impact, while 17.9% experienced a mild decline. Decline in sexual quality of life was significantly linked to religion, marital status, and education (*p* < 0.05). A majority of unskilled women (55%) experienced a decline in vasomotor-related quality of life, with a significant association (*p* < 0.001). The majority across all working groups reported no significant sexual health-related decline in quality of life (Table 3).

### 3.4 Work ability

Workability assessment revealed that 47.9% of women had moderate workability, 40.4% poor, 11.4% good, and only 0.4% excellent. Poor work ability was more common among women aged 45–59 years, those in nuclear families, and those in lower or middle socioeconomic classes (*p* < 0.001). Among unskilled women with moderate to severe menopausal symptoms, 39.5% reported poor work ability, whereas none of the symptom-free women did so-a difference that was statistically significant (*p* < 0.001). Conversely, in the semi-skilled/skilled and professional categories, work ability scores did not vary significantly across symptom levels. This highlights how physically demanding or low-autonomy jobs may worsen the impact of menopausal symptoms on day-to-day work performance (Table 4).

**Table 4:** Association between menopausal symptoms with work ability across occupation (*N* = 280).

Menopausal symptoms across occupations	Work ability *n* (%)		Chi square	*p* value
	Poor (*n*-113)	Good (*n*-167)		
Unskilled (*n* = 129)				
None	0 (0)	20 (15.5)	34.30	**0**.**00**
Mild	2 (1.6)	20 (15.5)		
Moderate to severe	51 (39.5)	36 (27.9)		
Semiskilled and skilled (*n* = 59)				
None	10 (16.9)	7 (11.9)	0.846	0.655
Mild	5 (8.5)	7 (11.9)		
Moderate to severe	15 (25.4)	15 (25.4)		
Clerical/Farmer/Shop owner/Profession (*n* = 92)				
None	8 (8.7)	12 (13.0)	1.100	0.590
Mild	7 (7.6)	12 (13.0)		
Moderate to severe	15 (16.3)	38 (41.3)		

Association between severity of menopausal symptoms and work ability across occupational categories was assessed using the Chi-square test.

Work ability was categorized as poor and good based on the Work Ability Index (WAI) scoring criteria.

Values are presented as frequency (*n*) with percentages in parentheses, calculated within each occupational group.

Bold values indicate statistically significant results (*p* < 0.05).

### 3.5 Correlation between menopausal symptoms, quality of life and work ability

#### 3.5.1 Correlation between menopausal symptoms and quality of life by occupation

**Figure 2:**
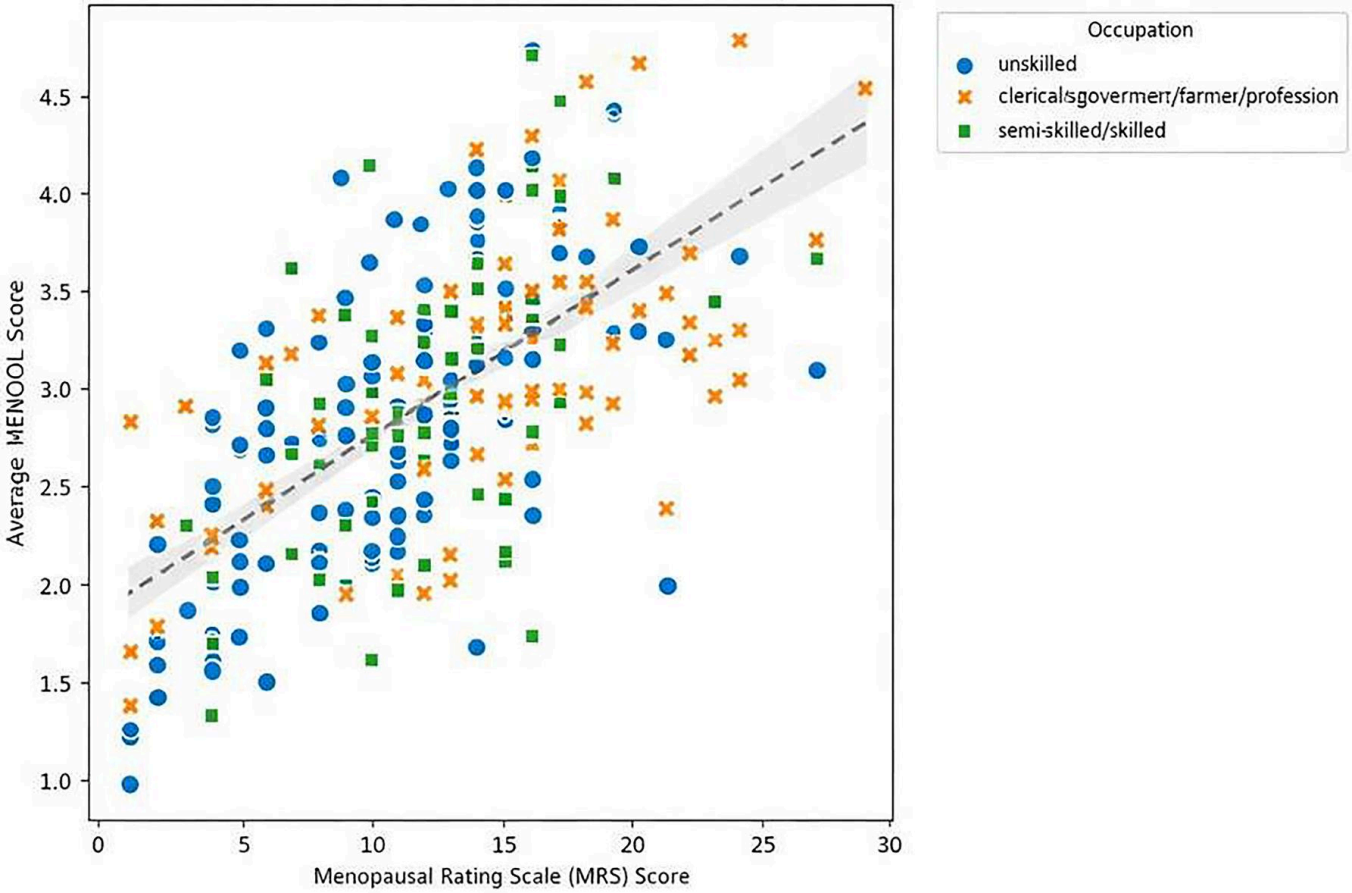
Correlation between menopausal symptoms (MRS) and quality of life by occupation.

Figure 2 shows a strong positive correlation between menopausal symptoms and their impact on overall quality of life was observed among both unskilled workers and clerical/professional workers (r = 0.685 and 0.68, respectively), while a moderate correlation (r = 0.52) was noted among semi-skilled/skilled workers. The dashed line reflects the overall trend across all occupational groups. Despite the clear relationship, the distribution of data points indicates substantial individual variation within each group.

#### 3.5.2 Correlation between menopausal symptoms and work ability by occupation

**Figure 3:**
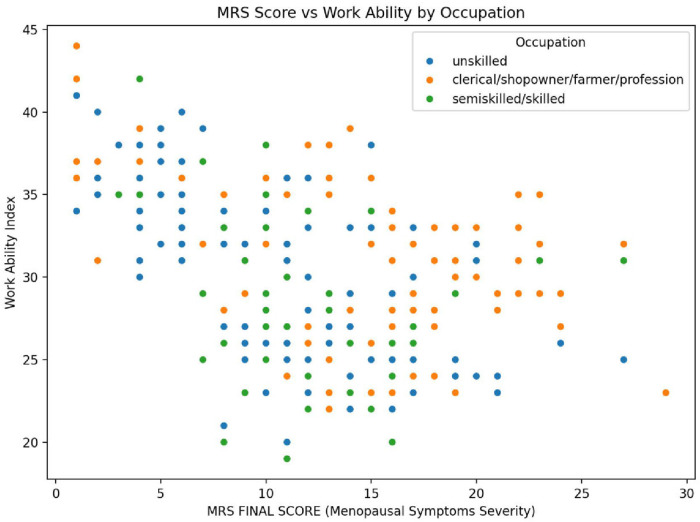
Correlation between menopausal symptoms and work ability across occupation (*N* = 280).

The scatter plot (Figure 3) shows that, as menopausal symptoms increase (higher MRS), work ability tends to decrease, but this relationship varies by occupation. This is noticed more in unskilled workers, who commonly report limited job abilities even with moderate symptoms. Although not as significantly, semi-skilled and skilled workers exhibit a similar tendency. In contrast, women in professional or clerical jobs seem to handle symptoms better, often maintaining good work ability. The decline was notably strong among unskilled women, reinforcing their increased vulnerability.

#### 3.5.3 Correlation matrix showing the relationship between menopausal symptoms, MENQOL domains, and work ability across occupational groups

**Figure 4:**
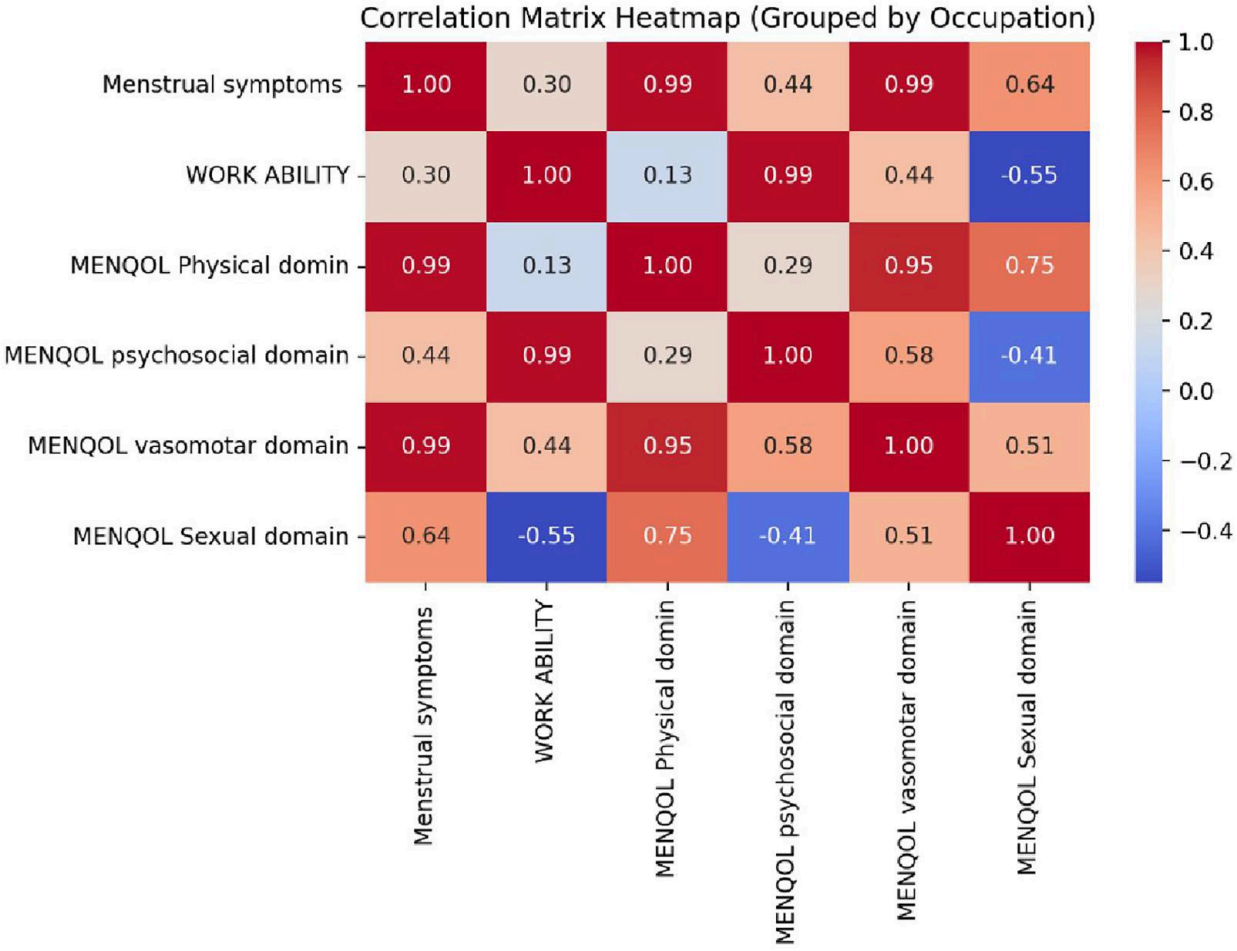
Correlation matrix between menopausal symptoms and quality-of-life domains (MENQOL), along with work ability, among different occupational groups.

The correlation matrix heat map grouped by occupation illustrates significant relationships between menopausal symptoms, quality of life, and work ability among employed women. Menopausal symptoms demonstrate very strong positive correlations with MENQOL physical (r = 0.99), vasomotor (r = 0.99), and psychosocial (r = 0.99) domains, indicating that heightened symptom severity markedly impairs overall quality of life. The physical domain further connects substantially with vasomotor (r = 0.95) and sexual (r = 0.75) domains, whereas work ability exhibits negative relationships with sexual (r = −0.55) and psychosocial (r = −0.29) domains, plus a weak positive link to the physical domain (r = 0.13). These findings underscore how menopausal symptoms disrupt physical, emotional, and sexual health, ultimately compromising occupational performance (Figure 4).

### 3.6 Predictors of low workability

Among employed menopausal women, bootstrap-adjusted multivariable logistic regression (400 replications) identified several symptoms and background characteristics as significant predictors of low work ability (WAI ≤27). In multivariable logistic regression analysis, women reporting reduced ability to accomplish usual activities had nearly two-fold higher odds of poor work ability (adjusted OR = 1.94, 95% CI: 1.22–2.97, *p*- 0.004). Sleep problems (adjusted OR = 1.90, 95% CI: 1.00–2.48, *p*- 0.048) and feeling tired or worn out (adjusted OR = 1.69, 95% CI: 1.12–2.34, *p*- 0.012) were also independently associated with poor work ability. Bootstrap validation demonstrated high stability for the key symptom predictors, supporting the robustness of the model (Table 5).

**Table 5:** Multivariable predictors of low work ability (WAI ≤ 27) among the employed menopausal women (*N* = 280).

Predictors	Adjusted OR	95% CI	*p*-value
Accomplishing less than I used to	**1**.**94**	**1.22**–**2.97**	**0**.**004**
Sleep problems (difficulty falling asleep)	**1**.**90**	**1.00**–**2.48**	**0**.**048**
Feeling tired or worn out	**1**.**69**	**1.12**–**2.34**	**0**.**012**
Dissatisfied with personal life	1.44	0.87–2.27	0.158
Age group (older vs. younger)	1.44	0.84–2.09	0.183
Vaginal dryness during intercourse	1.44	0.87–2.10	0.154
Aches in back, neck, or head	1.33	0.88–2.09	0.176
Hot flushes or flashes	1.30	0.76–1.73	0.319
Irritability	1.30	0.71–1.89	0.363
Dryness of vagina	1.29	0.85–1.84	0.214
Drying skin	1.27	0.70–1.63	0.401
Anxiety	1.27	0.73–1.92	0.374
Family income	1.26	0.78–1.68	0.342
Sexual problems	1.24	0.81–1.78	0.287
Decrease in stamina	1.17	0.91–1.47	0.201
Feeling impatient	1.16	0.73–1.81	0.489
Lack of energy	1.15	0.82–1.66	0.371
Family size	1.15	0.82–1.91	0.355
Involuntary urination	1.13	0.75–1.75	0.540
Heart discomfort	1.13	0.71–1.88	0.565
Decrease in physical strength	1.09	0.61–1.79	0.748
Socioeconomic status	1.08	0.75–1.77	0.680
Night sweats	1.03	0.71–1.65	0.892
Feeling of wanting to be alone	1.00	0.62–1.60	0.998
Aching muscles and joints	0.97	0.76–1.42	0.801
Marital status	0.95	0.55–1.69	0.873
Low backache	0.79	0.64–1.32	0.598
Participant's occupation	0.69	0.45–1.03	0.067
Change in sexual desire	0.65	0.50–1.25	0.071
Weight gain	0.63	0.40–1.04	0.069
Depressive mood	0.60	0.42–1.07	0.092
Sick leave during past year	0.46	0.35–0.72	<0.001
Work ability vs. job demands	0.38	0.23–0.63	<0.001
Estimated work impairment due to disease	0.15	0.11–0.31	<0.001
Prognosis of work ability (2 years)	0.15	0.11–0.25	<0.001
Current work ability vs. lifetime best	0.08	0.07–0.20	<0.001

OR, odds ratio; CI, confidence interval, Outcome was low WA1 score (≤27). Estimates were obtained using bootstrap logistic regression (400 resamples) with FDR-adjusted *p*-values;

Reference categories: Reference categories: For Menopause symptoms: Absence of the symptoms, Age group: younger age group.

Bold values indicate statistically significant results (*p* < 0.05).

## 4 Discussion

The present study demonstrates that menopausal symptoms are highly prevalent among employed women in southern Karnataka and are closely linked to decrements in both quality of life and work ability, with particularly adverse effects among women in unskilled occupations. Nearly three-quarters of participants in the present study reported moderate to severe symptoms on the Menopause Rating Scale. This is comparable to studies from southern India, where most women likewise fell within the moderate-to-severe symptom range (17, 30). This highlights that menopause is not merely a biological transition but a challenging phase affecting both personal and professional spheres.

The most common issues were psychosocial symptoms, which were followed by physical symptoms such as joint and muscle discomfort, irritation, sleep disruptions, and vasomotor complaints. Urogenital symptoms were the least reported symptoms among the participants. This burden is comparable to, or slightly higher than, estimates from recent community-based studies in India and other low- and middle-income settings, which also identify somatic and musculoskeletal complaints as dominant symptoms (31–33). For example, a 2024 meta-analysis of global menopausal symptom prevalence reported musculoskeletal pain in approximately two-thirds of midlife women, with the highest symptom burden in low-income regions, supporting the high levels observed in this cohort (11). Despite experiencing severe menopausal symptoms, most women do not seek any healthcare services (34).

Consistent with prior work, vasomotor and psychosocial symptoms in this study translated into meaningful impairments in day-to-day functioning and perceived quality of life. Over three quarters of women reported at least a mild decline in physical quality of life, and more than half reported psychosocial deterioration, patterns that mirror findings of MENQOL based studies where vasomotor, somatic and psychological domains contribute most strongly to reduced health related quality of life (35, 36). Nappi et al. similarly documented the substantial quality-of-life burden of vasomotor symptoms among European women, with fatigue and sleep problems playing a central role, which parallels the prominence of tiredness and sleep disturbance among the participants (7). These findings reinforce the view that menopausal transition should be considered a key determinant of midlife women's physical and mental well-being rather than a purely biological milestone.

A distinctive contribution of this study is the explicit examination of occupational differences. Unskilled workers showed the highest prevalence of moderate to severe menopausal symptoms and were significantly more likely to report declines in physical and psychosocial quality of life and poor work ability compared with semi-skilled, skilled and professional groups. These gradients by occupation align with international evidence that physically demanding, low-control jobs amplify the impact of menopausal symptoms on work outcomes (19, 37, 38). In the Health and Employment After Fifty (HEAF) cohort from the UK, for example, women with severe symptoms were more likely to reduce working hours, change jobs or contemplate leaving employment, with musculoskeletal pain, fatigue and sleep problems strongly associated with work impairment (6). According to the study's findings, similar dynamics might be at play in India, where a large number of unskilled professions need manual labour, little autonomy, and little workplace flexibility, which would increase the functional impact of symptoms like weariness and joint discomfort (17).

Work ability emerged as a critical outcome in this cohort: almost forty percent of women had poor work ability, and only a small minority reported good or excellent scores on the WAI. The strong association between higher symptom burden and poorer workability, particularly among unskilled workers, is consistent with Dutch and Scandinavian studies showing that severe menopausal symptoms independently predict reduced work ability and sickness absence even after adjustment for age, comorbidity and job demands (20, 37, 39). Correlation analyses further indicate that symptom severity is linked not only to global work ability, but also to specific domains such as perceived physical capacity and mental resources, underscoring the multidimensional nature of the impact. Together, these findings support the concept that menopause is an important, although largely invisible, occupational health concern in medium income settings.

An interesting feature of the data is that, despite high levels of somatic and psychosocial symptoms, the sexual domain of quality of life appeared less affected, with the majority reporting no or only mild decline. While this could reflect genuinely lower sexual morbidity, it is more plausibly explained by under-reporting due to stigma and discomfort in discussing sexual concerns, as highlighted in previous Indian studies of menopausal women. Qualitative work from multiple cultural contexts reveals that societal norms, lack of privacy and limited provider participation around sexual health often discourage open disclosure, which may lead to underestimating of sexual issues in structured questionnaires (40–42). When assessing the seemingly minor effect of menopause on sexual well-being among the study participants, this potential reporting bias should be taken into consideration.

The correlation analyses revealed strong positive associations between menopausal symptom severity (MRS scores) and declines in quality of life across occupational groups. These findings align with prior studies using MRS and MENQOL, which report moderate-to-strong positive correlations between symptom severity and impaired physical, vasomotor, and psychosocial QoL domains, particularly in working women from LMICs (43). The steeper trend among unskilled workers underscores occupational vulnerability, consistent with evidence that physically demanding roles amplify symptom impact on daily functioning.

The correlation matrix confirmed near-perfect inter-domain linkages within MENQOL alongside moderate negative ties to WAI. Such patterns validate MENQOL's structure, where vasomotor/physical symptoms drive 66%–76% QoL impairment, often spilling into work via reduced self-efficacy (44, 45). Weak positive physical-WAI association may reflect adaptive coping in resilient groups, though overall data highlight menopause as a modifiable barrier to workforce retention in India.

The current findings show that few menopausal symptoms independently predict low work ability among employed women in southern India. The strong link to reduced daily functioning highlights menopause as a modifiable occupational barrier, particularly in India's ageing female workforce, where 37% of women in their 50s are still active. Sleep issues and exhaustion, which affect more than 40% of the global population, are associated with cohorts where vasomotor/sleep disturbances cause absenteeism through cognitive impairment and fatigue. Bootstrap stability across iterations validates reliability, showing mechanistic pathways: night sweats impair sleep architecture, exacerbating daytime weariness and deconditioning in inflexible unskilled employment. To reduce attrition in low- and middle-income countries, this symptom cluster requires the integration of occupational screening, sleep hygiene, and flexible scheduling (46–49).

These results extend prior evidence by quantifying occupational risk in a low-resource context, where unskilled workers face compounded physical demands. The protective effect of rural residence and larger households may reflect greater social support and physical autonomy compared to urban settings, though reverse causality cannot be excluded in this cross-sectional design. Many rural women engaged in unskilled occupations also juggle multiple job roles to meet urban market demands. Unexpectedly, higher education increased low work ability risk, possibly indicating greater symptom awareness or a mismatch between cognitive demands and menopausal cognitive fog, warranting prospective confirmation (50).

Workplace regulations should promote menopause assistance through flexible hours, wellness spaces, and awareness initiatives tailored to unskilled workers carrying the biggest symptom burden. Incorporate routine menopause screening and counselling into Health and Wellness Centres, and train ASHA/ANM/CHO staff to handle symptoms compassionately. National recommendations must acknowledge menopause as an occupational health priority to boost midlife women's productivity and retention (51).

This study contributes to the UN Sustainable Development Goals (SDGs) by addressing SDG 3 (Good Health and Well-being) through evidence that menopause is an underappreciated non-communicable disease risk factor impacting a significant proportion of working women, SDG 5 (Gender Equality) by emphasising occupational disparities and stigma surrounding women's reproductive health, SDG 8 (Decent Work) by identifying menopause-related work impairment, and SDG 10 (Reduced Inequalities) through urban-rural and occupational stratification that informs inclusive workplace policies for midlife women in low- and middle-income countries (LMICs) (51).

### 4.1 Strengths and limitations

This study offers a fresh and comprehensive look at how menopause impacts physical health, quality of life, and work ability among employed women. The use of standardised questionnaires like as MRS, MENQOL, and WAI provides a thorough understanding of the effects of menopause on women's life. Face-to-face interviews encouraged candid discussions, including sensitive themes. Furthermore, the bootstrap resampling (400 iterations) and FDR correction gave strong evidence against multiple testing and Type I error inflation.

However, certain limitations must be acknowledged. The study's cross-sectional design prevents causal inference; whereas more severe symptoms were linked to lower quality of life and work performance, temporal associations could not be determined. Occupational categories covered a wide range of duties and responsibilities, obscuring differences in autonomy and physical exertion. This heterogeneity reduces the ability to discover subtle impacts of job autonomy and physical demands; future studies should use more extensive job exposure matrices to account for these disparities. The data on menopausal symptoms, quality of life, and job performance were self-reported and could be influenced by recollection or social desirability bias. Cultural norms surrounding modesty and stigma, particularly in terms of sexual health, may have contributed to underreporting in the urogenital and sexual domains. The study used a relatively small sample of women drawn from a single district in southern India, which may limit the findings' applicability to other places. Likewise, while probability sampling was impractical due to absent sampling frames for informal workers, multistage purposive recruitment within stratified urban/rural clusters ensured targeted access to eligible postmenopausal workers. Selection bias was minimized through systematic house-to-house screening, uniform eligibility criteria, supervisor oversight, and sensitivity analyses confirming outcome consistency across strata (*χ*^2^ *p* > 0.05), though single-district focus limits broader generalisability. The bootstrap analysis (400 resamples) produced reliable estimates, but smaller iterations risk instability. Finally, unmeasured variables such as comorbid chronic diseases, workplace policies, and supervisor assistance were not adequately captured, potentially confounding certain correlations.

### 4.2 Future research

Longitudinal study designs are needed to assess how symptoms, job abilities, and employment trajectories change over time, and whether improvements in workplace environment or access to care can ameliorate negative effects. Mixed methods approaches involving in-depth interviews or focus groups with workers, employers, and health care providers might aid in unravelling the mechanisms underlying the quantitative patterns identified here. These mechanisms include stigma, coping strategies, and organisational culture.

## 5 Conclusion

This community-based cross-sectional study reveals that a substantial proportion of employed postmenopausal women in urban-rural Mysuru experienced moderate-to-severe menopausal symptoms, with joint/muscle pain, sleep disturbances, and vasomotor complaints predominant, causing significant declines in physical QoL, psychosocial functioning, and work ability - particularly among unskilled workers facing physically demanding roles. Women involved in unskilled work demonstrated heightened vulnerability, compared to their semi-skilled and skilled counterparts, with a stronger symptom burden, poorer work ability, and greater impairment in menopause-related quality of life. The strong association between menopausal symptom severity and reduced work ability highlights how occupational demands intensify the impact of menopause in low- and middle-income settings, where many older women remain economically active despite limited workplace support.

## Data Availability

The raw data supporting the conclusions of this article will be made available by the authors, without undue reservation.
