# Correction: Menopausal symptoms, work ability, and quality of life: a cross- sectional study among employed women in southern India

**DOI:** 10.3389/fgwh.2026.1867821

**Published:** 2026-05-27

**Authors:** Frontiers Production Office

**Affiliations:** Frontiers Media SA, Lausanne, Switzerland

**Keywords:** employed women, India, menopausal symptoms, menopausal women, menopause rating scale, quality of life, work ability

The reference 51 was erroneously written as “51. United Nations. The 17 sustainable development goals. United Nations (2015). Available online at: https://sdgs.un.org/goals (Accessed May 02, 2026)”. It should be “51. United Nations. The 17 sustainable development goals. United Nations (2015). Available online at: https://sdgs.un.org/goals (Accessed February 05, 2026)”.

The original version of this article has been updated.

